# 1,1-Bis(4-fluoro­phen­yl)-3,4-dihydro-1*H*-1,3-oxazino[3,4-*a*]indole

**DOI:** 10.1107/S160053680803376X

**Published:** 2008-10-31

**Authors:** Weijun Fu, Dongsheng Deng, Dongfeng Hong, Zhiqiang Wang, Baoming Ji

**Affiliations:** a College of Chemistry and Chemical Engineering, Luoyang Normal University, Luoyang 471022, People’s Republic of China

## Abstract

The title compound, C_23_H_17_F_2_NO, which crystallizes with two independent mol­ecules in the asymmetric unit, was prepared by the cyclization of 4-[2-bis­(4-fluoro­phen­yl)methyl­eneamino]but-3-yn-1-ol at room temperature. The mol­ecules display a tripod conformation. The two fluoro­phenyl rings make dihedral angles of 79.26 (2) and 85.87 (1)° [86.53 (1) and 83.67 (2)° in the second mol­ecule] with the indole ring, and the dihedral angles between the fluoro­phenyl rings are 67.74 (2) and 66.33 (2)°, respectively. Furthermore, the indole rings are located on the edge of the respective oxazine half-chair ring systems. Nonconventional C—H⋯π contacts between indole and fluoro­phenyl rings are observed.

## Related literature

For bond-length and angle data, see: Lee *et al.* (2000 ▶). For the synthesis of palladium-catalysed key inter­mediates to condensed heteroaromatic rings, see: Sakamoto *et al.* (1988 ▶). For the biological function of tryptamines and their derivatives, see: Monckton & McCormick (2002 ▶).
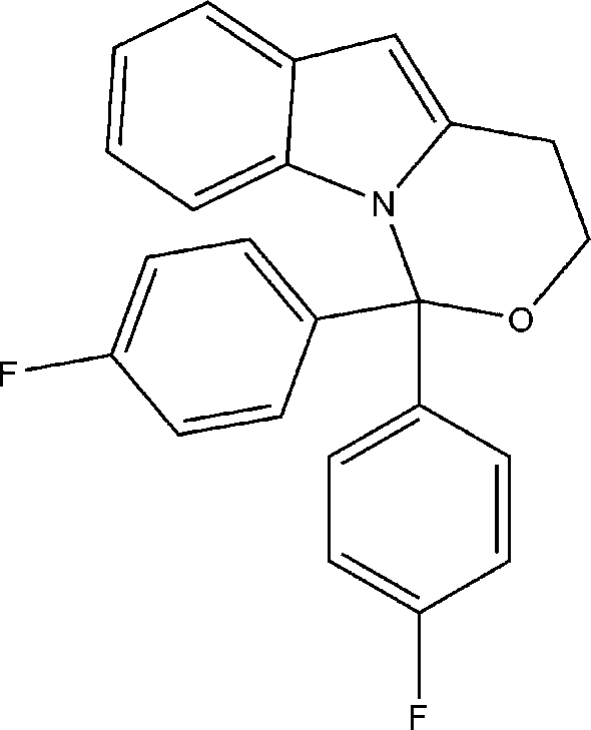


## Experimental

### 

#### Crystal data

C_23_H_17_F_2_NO
*M* *_r_* = 361.38
Triclinic, 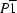

*a* = 10.9845 (11) Å
*b* = 11.2002 (11) Å
*c* = 15.1964 (16) Å
α = 87.6150 (10)°
β = 80.7510 (10)°
γ = 76.9900 (10)°
*V* = 1797.9 (3) Å^3^
*Z* = 4
Mo *K*α radiation
μ = 0.10 mm^−1^
*T* = 291 (2) K
0.38 × 0.30 × 0.20 mm

#### Data collection

Bruker APEXII CCD diffractometer
Absorption correction: multi-scan (*SADABS*; Sheldrick, 1996 ▶) *T*_min_ = 0.965, *T*_max_ = 0.981
13827 measured reflections
6630 independent reflections
4487 reflections with *I* > 2σ(*I*)
*R*_int_ = 0.019

#### Refinement

*R*[*F* ^2^ > 2σ(*F* ^2^)] = 0.039
*wR*(*F* ^2^) = 0.106
*S* = 1.02
6630 reflections
487 parameters
H-atom parameters constrained
Δρ_max_ = 0.13 e Å^−3^
Δρ_min_ = −0.16 e Å^−3^

### 

Data collection: *APEX2* (Bruker, 2004 ▶); cell refinement: *SAINT* (Bruker, 2004 ▶); data reduction: *SAINT*; program(s) used to solve structure: *SHELXS97* (Sheldrick, 2008 ▶); program(s) used to refine structure: *SHELXL97* (Sheldrick, 2008 ▶); molecular graphics: *SHELXTL* (Sheldrick, 2008 ▶); software used to prepare material for publication: *SHELXTL* and *PLATON* (Spek, 2003 ▶).

## Supplementary Material

Crystal structure: contains datablocks global, I. DOI: 10.1107/S160053680803376X/si2115sup1.cif

Structure factors: contains datablocks I. DOI: 10.1107/S160053680803376X/si2115Isup2.hkl

Additional supplementary materials:  crystallographic information; 3D view; checkCIF report

## Figures and Tables

**Table 1:** Hydrogen-bond geometry (Å, °) *Cg*3, *Cg*5, *Cg*9 and *Cg*11 are the centroids of the C3–C8, C26–C31, N2/C24–C26/C31 and C18–C23 rings, respectively.

*D*—H⋯*A*	*D*—H	H⋯*A*	*D*⋯*A*	*D*—H⋯*A*
C14—H14⋯*Cg*3^i^	0.93	2.88	3.769 (2)	159
C29—H29⋯*Cg*5^ii^	0.93	2.93	3.747 (2)	148
C43—H43⋯*Cg*11^iii^	0.93	2.79	3.695 (2)	165
C40—H40⋯*Cg*9	0.93	2.74	3.3790 (7)	127

Symmetry codes: (i) 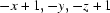; (ii) 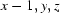; (iii) 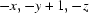.

**Fig. 1.**
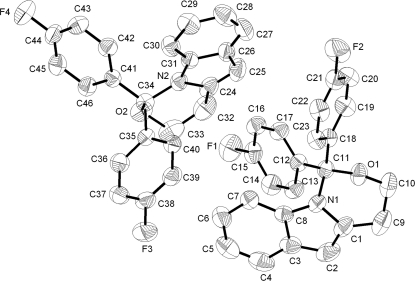
View of the molecular structure of (I) with atom numbering scheme and 30% probability displacement ellipsoids for non-hydrogen atoms.

## supplementary crystallographic information

### Comment

Indoles are one of the most widely distributed heterocyclic compounds in nature (Sakamoto *et al.*, 1988). The indole ring appeares in tryptophan, an essential amino acid, and metabolites of tryptophan are important in the biological chemistry of both plants and animals (Monckton & McCormick, 2002). The potent physiological properties of these indole derivatives led to vast research of their use as medicines in the field of pharmaceutical chemistry. So in the recent decades, many chemists have been attracted by the synthesis of indoles. In this context, we report the synthesis of the title compound.

The asymmetric unit of the title compound contains two independent molecules (Fig. 1.), in both of which the bond lengths and angles are within ranges as reported by Lee *et al.* (2000). The structural analysis reveals that a surprizing feature is the lack of C—H···F interactions, but three weak non-conventional intermolecular C—H···π contacts with two fluorophenyl rings (F1, F4) and one indole ring are donors, whereas the indole rings (C3 - C8 and C26 - C31) and a fluorophenyl ring (C18 - C23) are π acceptors. Cg3, Cg5 and Cg11 are the centroids of the acceptor rings, for the intramolecular C—H···π contact, Cg9 is the centroid of the five-membered ring (N2, C24, C25, C26, C31). Details are given in Table 1. Furthermore, pseudosymmetry between the independent molecules can be described as an approximate twofold screw axis when viewed down the *a* axis.

### Experimental

To a solution of 4-(2-bis(4-fluoridophenyl)methyleneamino)phenyl)but-3-yn-1-ol (0.5 mmol) in dry CH_2_Cl_2_ was added AuCl_3_ (5 mg). The mixture was stirred for 1 h at room temperature. After evaporation of the solvent, the residue was purified by column chromatography on silica gel(petroleum ether) to afford the title compound as a colorless solid (163 mg, yield 90%). The title compound was recrystallized from petroleum ether at room temperature to give the desired crystals suitable for single-crystal X-ray diffraction.

### Refinement

All H atoms were positioned geometrically and treated as riding, with C—H bond lengths constrained to 0.93 Å (aromatic CH) or 0.97 Å (methylene CH_2_), and with *U*ĩso~(H) = 1.2Ueq(C) or 1.5Ueq(methylene C).

### Crystal data

**Table:**

C_23_H_17_F_2_NO	*Z* = 4
*M**_r_* = 361.38	*F*(000) = 752
Triclinic, *P*1	*D*_x_ = 1.335 Mg m^−^^3^
Hall symbol: -P 1	Mo *K*α radiation, λ = 0.71073 Å
*a* = 10.9845 (11) Å	Cell parameters from 3583 reflections
*b* = 11.2002 (11) Å	θ = 2.3–23.5°
*c* = 15.1964 (16) Å	µ = 0.10 mm^−^^1^
α = 87.615 (1)°	*T* = 291 K
β = 80.751 (1)°	Block, yellow
γ = 76.990 (1)°	0.38 × 0.30 × 0.20 mm
*V* = 1797.9 (3) Å^3^	

### Data collection

**Table:**

Bruker APEXII CCD diffractometer	6630 independent reflections
Radiation source: fine-focus sealed tube	4487 reflections with *I* > 2σ(*I*)
graphite	*R*_int_ = 0.019
φ and ω scans	θ_max_ = 25.5°, θ_min_ = 2.3°
Absorption correction: multi-scan (SADABS; Sheldrick, 1996)	*h* = −13→13
*T*_min_ = 0.965, *T*_max_ = 0.981	*k* = −13→12
13827 measured reflections	*l* = −18→18

### Refinement

**Table:**

Refinement on *F*^2^	Primary atom site location: structure-invariant direct methods
Least-squares matrix: full	Secondary atom site location: difference Fourier map
*R*[*F*^2^ > 2σ(*F*^2^)] = 0.039	Hydrogen site location: inferred from neighbouring sites
*wR*(*F*^2^) = 0.106	H-atom parameters constrained
*S* = 1.02	*w* = 1/[σ^2^(*F*_o_^2^) + (0.0461*P*)^2^ + 0.2061*P*] where *P* = (*F*_o_^2^ + 2*F*_c_^2^)/3
6630 reflections	(Δ/σ)_max_ < 0.001
487 parameters	Δρ_max_ = 0.13 e Å^−^^3^
0 restraints	Δρ_min_ = −0.16 e Å^−^^3^

### Special details

**Table:**

Geometry. All e.s.d.'s (except the e.s.d. in the dihedral angle between two l.s. planes) are estimated using the full covariance matrix. The cell e.s.d.'s are taken into account individually in the estimation of e.s.d.'s in distances, angles and torsion angles; correlations between e.s.d.'s in cell parameters are only used when they are defined by crystal symmetry. An approximate (isotropic) treatment of cell e.s.d.'s is used for estimating e.s.d.'s involving l.s. planes.
Refinement. Refinement of *F*^2^ against ALL reflections. The weighted *R*-factor *wR* and goodness of fit *S* are based on *F*^2^, conventional *R*-factors *R* are based on *F*, with *F* set to zero for negative *F*^2^. The threshold expression of *F*^2^ > σ(*F*^2^) is used only for calculating *R*-factors(gt) *etc*. and is not relevant to the choice of reflections for refinement. *R*-factors based on *F*^2^ are statistically about twice as large as those based on *F*, and *R*- factors based on ALL data will be even larger.

### Fractional atomic coordinates and isotropic or equivalent isotropic displacement parameters (Å^2^)

**Table:**

	*x*	*y*	*z*	*U*_iso_*/*U*_eq_	
F1	0.15332 (10)	0.20474 (12)	0.53431 (8)	0.0843 (4)	
F2	0.62785 (13)	0.67584 (11)	0.14682 (8)	0.0878 (4)	
F3	0.30250 (12)	−0.18113 (10)	0.34221 (7)	0.0807 (4)	
F4	−0.23605 (11)	0.27910 (12)	−0.03884 (8)	0.0839 (4)	
O1	0.66525 (11)	0.33741 (11)	0.48340 (7)	0.0549 (3)	
O2	0.31522 (11)	0.17610 (11)	0.01727 (7)	0.0549 (3)	
N1	0.72344 (12)	0.18387 (13)	0.37478 (9)	0.0466 (3)	
N2	0.24016 (12)	0.32408 (12)	0.12899 (9)	0.0451 (3)	
C1	0.84755 (16)	0.15765 (18)	0.39117 (12)	0.0581 (5)	
C2	0.91322 (18)	0.05810 (19)	0.34339 (14)	0.0707 (6)	
H2	0.9981	0.0217	0.3433	0.085*	
C3	0.83131 (16)	0.01843 (16)	0.29349 (12)	0.0549 (5)	
C4	0.8460 (2)	−0.07701 (18)	0.23367 (15)	0.0727 (6)	
H4	0.9241	−0.1311	0.2196	0.087*	
C5	0.7454 (2)	−0.09050 (19)	0.19602 (14)	0.0744 (6)	
H5	0.7551	−0.1540	0.1561	0.089*	
C6	0.6289 (2)	−0.01063 (18)	0.21662 (13)	0.0661 (5)	
H6	0.5613	−0.0222	0.1906	0.079*	
C7	0.61015 (17)	0.08564 (16)	0.27459 (12)	0.0543 (5)	
H7	0.5319	0.1397	0.2872	0.065*	
C8	0.71239 (15)	0.09884 (15)	0.31342 (11)	0.0447 (4)	
C9	0.8840 (2)	0.2312 (2)	0.45833 (15)	0.0774 (6)	
H9A	0.9683	0.2442	0.4376	0.093*	
H9B	0.8858	0.1860	0.5141	0.093*	
C10	0.79304 (19)	0.3529 (2)	0.47394 (13)	0.0694 (6)	
H10A	0.8057	0.3896	0.5275	0.083*	
H10B	0.8085	0.4074	0.4242	0.083*	
C11	0.63226 (15)	0.29876 (15)	0.40412 (10)	0.0436 (4)	
C12	0.50071 (15)	0.27351 (15)	0.43494 (10)	0.0418 (4)	
C13	0.48805 (17)	0.18616 (16)	0.50129 (11)	0.0511 (4)	
H13	0.5596	0.1425	0.5232	0.061*	
C14	0.37155 (18)	0.16283 (17)	0.53536 (12)	0.0569 (5)	
H14	0.3637	0.1042	0.5798	0.068*	
C15	0.26817 (17)	0.22808 (17)	0.50214 (12)	0.0542 (5)	
C16	0.27549 (17)	0.31452 (17)	0.43676 (12)	0.0554 (5)	
H16	0.2033	0.3574	0.4153	0.066*	
C17	0.39377 (16)	0.33680 (16)	0.40305 (11)	0.0498 (4)	
H17	0.4008	0.3953	0.3583	0.060*	
C18	0.63506 (15)	0.39877 (15)	0.33247 (10)	0.0425 (4)	
C19	0.57976 (17)	0.51909 (17)	0.35633 (12)	0.0564 (5)	
H19	0.5432	0.5370	0.4152	0.068*	
C20	0.57780 (18)	0.61303 (17)	0.29443 (13)	0.0595 (5)	
H20	0.5407	0.6937	0.3109	0.071*	
C21	0.63185 (18)	0.58419 (18)	0.20835 (12)	0.0568 (5)	
C22	0.68926 (18)	0.46804 (17)	0.18220 (12)	0.0569 (5)	
H22	0.7267	0.4515	0.1233	0.068*	
C23	0.69101 (16)	0.37452 (16)	0.24493 (11)	0.0488 (4)	
H23	0.7303	0.2945	0.2280	0.059*	
C24	0.35721 (16)	0.35631 (18)	0.11829 (12)	0.0542 (5)	
C25	0.34808 (19)	0.45452 (19)	0.16917 (13)	0.0644 (5)	
H25	0.4132	0.4935	0.1736	0.077*	
C26	0.22186 (17)	0.48855 (16)	0.21507 (11)	0.0513 (4)	
C27	0.1581 (2)	0.58283 (18)	0.27414 (13)	0.0673 (6)	
H27	0.2007	0.6382	0.2921	0.081*	
C28	0.0323 (2)	0.59277 (19)	0.30526 (13)	0.0721 (6)	
H28	−0.0109	0.6558	0.3440	0.087*	
C29	−0.0313 (2)	0.50920 (18)	0.27940 (13)	0.0652 (5)	
H29	−0.1169	0.5180	0.3010	0.078*	
C30	0.02879 (16)	0.41391 (16)	0.22286 (12)	0.0546 (5)	
H30	−0.0143	0.3574	0.2073	0.065*	
C31	0.15591 (15)	0.40480 (14)	0.18968 (10)	0.0426 (4)	
C32	0.46405 (18)	0.2895 (2)	0.05241 (14)	0.0743 (6)	
H32A	0.5423	0.2783	0.0767	0.089*	
H32B	0.4719	0.3381	−0.0017	0.089*	
C33	0.44347 (17)	0.1671 (2)	0.03051 (13)	0.0697 (6)	
H33A	0.5004	0.1358	−0.0232	0.084*	
H33B	0.4625	0.1100	0.0787	0.084*	
C34	0.22400 (15)	0.20732 (15)	0.09558 (10)	0.0436 (4)	
C35	0.24538 (14)	0.10483 (15)	0.16475 (10)	0.0427 (4)	
C36	0.25806 (17)	−0.01527 (16)	0.13810 (12)	0.0552 (5)	
H36	0.2531	−0.0308	0.0793	0.066*	
C37	0.27782 (18)	−0.11157 (17)	0.19745 (12)	0.0597 (5)	
H37	0.2865	−0.1917	0.1793	0.072*	
C38	0.28430 (17)	−0.08643 (17)	0.28335 (12)	0.0538 (5)	
C39	0.27395 (17)	0.02883 (17)	0.31220 (12)	0.0556 (5)	
H39	0.2799	0.0429	0.3711	0.067*	
C40	0.25431 (16)	0.12532 (16)	0.25213 (11)	0.0490 (4)	
H40	0.2471	0.2049	0.2710	0.059*	
C41	0.09707 (15)	0.22610 (15)	0.06247 (10)	0.0421 (4)	
C42	0.07212 (17)	0.31129 (16)	−0.00514 (11)	0.0514 (4)	
H42	0.1315	0.3569	−0.0274	0.062*	
C43	−0.03962 (18)	0.32917 (17)	−0.03970 (12)	0.0576 (5)	
H43	−0.0562	0.3861	−0.0850	0.069*	
C44	−0.12525 (17)	0.26093 (18)	−0.00561 (12)	0.0549 (5)	
C45	−0.10527 (17)	0.17695 (17)	0.06081 (12)	0.0571 (5)	
H45	−0.1654	0.1320	0.0827	0.068*	
C46	0.00684 (16)	0.16015 (16)	0.09501 (11)	0.0517 (4)	
H46	0.0218	0.1035	0.1407	0.062*	

### Atomic displacement parameters (Å^2^)

**Table:**

	*U*^11^	*U*^22^	*U*^33^	*U*^12^	*U*^13^	*U*^23^
F1	0.0564 (7)	0.1096 (10)	0.0900 (9)	−0.0353 (7)	0.0025 (6)	0.0043 (7)
F2	0.1287 (11)	0.0711 (8)	0.0718 (8)	−0.0411 (7)	−0.0199 (7)	0.0276 (6)
F3	0.1046 (9)	0.0656 (8)	0.0735 (8)	−0.0146 (7)	−0.0313 (7)	0.0242 (6)
F4	0.0631 (7)	0.1079 (10)	0.0883 (8)	−0.0183 (7)	−0.0361 (6)	0.0022 (7)
O1	0.0619 (8)	0.0689 (8)	0.0405 (6)	−0.0249 (6)	−0.0122 (5)	−0.0023 (6)
O2	0.0485 (7)	0.0683 (8)	0.0417 (6)	−0.0058 (6)	0.0018 (5)	−0.0001 (6)
N1	0.0378 (8)	0.0530 (9)	0.0492 (8)	−0.0084 (6)	−0.0108 (6)	0.0025 (7)
N2	0.0389 (8)	0.0498 (9)	0.0487 (8)	−0.0151 (6)	−0.0060 (6)	0.0026 (7)
C1	0.0395 (10)	0.0734 (13)	0.0640 (12)	−0.0154 (9)	−0.0145 (9)	0.0116 (10)
C2	0.0364 (11)	0.0765 (15)	0.0919 (15)	−0.0001 (10)	−0.0083 (10)	0.0099 (12)
C3	0.0440 (10)	0.0498 (11)	0.0653 (12)	−0.0059 (8)	−0.0001 (9)	0.0067 (9)
C4	0.0637 (14)	0.0513 (12)	0.0888 (15)	0.0019 (10)	0.0100 (12)	−0.0017 (11)
C5	0.0895 (17)	0.0536 (13)	0.0748 (14)	−0.0141 (12)	0.0033 (12)	−0.0111 (11)
C6	0.0764 (14)	0.0593 (13)	0.0661 (13)	−0.0179 (11)	−0.0157 (10)	−0.0071 (10)
C7	0.0525 (11)	0.0519 (11)	0.0588 (11)	−0.0082 (9)	−0.0135 (9)	−0.0041 (9)
C8	0.0451 (10)	0.0413 (10)	0.0459 (9)	−0.0088 (8)	−0.0038 (7)	0.0046 (8)
C9	0.0566 (13)	0.1079 (19)	0.0782 (14)	−0.0296 (13)	−0.0264 (11)	0.0043 (13)
C10	0.0706 (14)	0.0911 (16)	0.0608 (12)	−0.0395 (12)	−0.0225 (10)	0.0013 (11)
C11	0.0467 (10)	0.0480 (10)	0.0371 (9)	−0.0110 (8)	−0.0083 (7)	−0.0018 (7)
C12	0.0438 (10)	0.0447 (10)	0.0363 (8)	−0.0095 (8)	−0.0050 (7)	−0.0012 (7)
C13	0.0499 (11)	0.0551 (11)	0.0483 (10)	−0.0109 (8)	−0.0107 (8)	0.0071 (8)
C14	0.0599 (12)	0.0615 (12)	0.0518 (11)	−0.0228 (10)	−0.0047 (9)	0.0095 (9)
C15	0.0424 (11)	0.0655 (12)	0.0563 (11)	−0.0206 (9)	0.0013 (8)	−0.0073 (10)
C16	0.0442 (11)	0.0588 (12)	0.0624 (12)	−0.0074 (9)	−0.0110 (9)	−0.0016 (9)
C17	0.0485 (11)	0.0541 (11)	0.0468 (10)	−0.0110 (8)	−0.0092 (8)	0.0064 (8)
C18	0.0427 (9)	0.0472 (10)	0.0391 (9)	−0.0143 (8)	−0.0042 (7)	−0.0016 (7)
C19	0.0656 (12)	0.0559 (12)	0.0445 (10)	−0.0131 (9)	0.0004 (9)	−0.0025 (9)
C20	0.0686 (13)	0.0464 (11)	0.0621 (12)	−0.0116 (9)	−0.0075 (10)	−0.0009 (9)
C21	0.0670 (12)	0.0585 (12)	0.0529 (11)	−0.0301 (10)	−0.0146 (9)	0.0170 (9)
C22	0.0660 (12)	0.0643 (13)	0.0430 (10)	−0.0277 (10)	0.0024 (8)	0.0006 (9)
C23	0.0515 (10)	0.0500 (11)	0.0450 (10)	−0.0170 (8)	0.0008 (8)	−0.0026 (8)
C24	0.0399 (10)	0.0705 (13)	0.0561 (11)	−0.0206 (9)	−0.0105 (8)	0.0131 (10)
C25	0.0587 (13)	0.0767 (14)	0.0719 (13)	−0.0374 (11)	−0.0227 (10)	0.0111 (11)
C26	0.0619 (12)	0.0502 (11)	0.0498 (10)	−0.0235 (9)	−0.0193 (9)	0.0097 (8)
C27	0.0999 (17)	0.0533 (12)	0.0590 (12)	−0.0316 (12)	−0.0225 (12)	0.0012 (10)
C28	0.0971 (17)	0.0557 (13)	0.0596 (12)	−0.0133 (12)	−0.0038 (12)	−0.0078 (10)
C29	0.0640 (13)	0.0629 (13)	0.0628 (12)	−0.0107 (10)	0.0038 (10)	−0.0055 (10)
C30	0.0518 (11)	0.0551 (11)	0.0574 (11)	−0.0175 (9)	−0.0010 (8)	−0.0056 (9)
C31	0.0453 (10)	0.0428 (10)	0.0414 (9)	−0.0131 (8)	−0.0088 (7)	0.0059 (7)
C32	0.0443 (12)	0.1052 (18)	0.0729 (14)	−0.0216 (11)	−0.0025 (10)	0.0066 (12)
C33	0.0412 (11)	0.0929 (16)	0.0624 (12)	0.0007 (10)	0.0055 (9)	0.0044 (11)
C34	0.0413 (9)	0.0474 (10)	0.0403 (9)	−0.0091 (8)	−0.0026 (7)	0.0001 (7)
C35	0.0381 (9)	0.0475 (10)	0.0423 (9)	−0.0084 (7)	−0.0077 (7)	0.0006 (8)
C36	0.0668 (12)	0.0527 (11)	0.0461 (10)	−0.0095 (9)	−0.0138 (9)	−0.0029 (9)
C37	0.0731 (13)	0.0449 (11)	0.0612 (12)	−0.0081 (9)	−0.0176 (10)	−0.0006 (9)
C38	0.0541 (11)	0.0534 (12)	0.0537 (11)	−0.0079 (9)	−0.0169 (8)	0.0129 (9)
C39	0.0619 (12)	0.0622 (13)	0.0446 (10)	−0.0103 (9)	−0.0194 (8)	0.0031 (9)
C40	0.0518 (10)	0.0483 (10)	0.0483 (10)	−0.0097 (8)	−0.0136 (8)	−0.0019 (8)
C41	0.0445 (10)	0.0443 (10)	0.0376 (9)	−0.0097 (8)	−0.0063 (7)	−0.0021 (7)
C42	0.0523 (11)	0.0521 (11)	0.0510 (10)	−0.0145 (8)	−0.0092 (8)	0.0074 (8)
C43	0.0587 (12)	0.0618 (12)	0.0510 (10)	−0.0069 (10)	−0.0166 (9)	0.0096 (9)
C44	0.0470 (11)	0.0640 (12)	0.0561 (11)	−0.0087 (9)	−0.0183 (9)	−0.0079 (10)
C45	0.0509 (11)	0.0636 (12)	0.0615 (12)	−0.0213 (9)	−0.0104 (9)	−0.0005 (10)
C46	0.0541 (11)	0.0553 (11)	0.0489 (10)	−0.0178 (9)	−0.0117 (8)	0.0078 (8)

### Geometric parameters (Å, °)

**Table:**

F1—C15	1.356 (2)	C19—H19	0.9300
F2—C21	1.3575 (19)	C20—C21	1.366 (2)
F3—C38	1.3595 (19)	C20—H20	0.9300
F4—C44	1.362 (2)	C21—C22	1.357 (3)
O1—C11	1.4229 (18)	C22—C23	1.386 (2)
O1—C10	1.437 (2)	C22—H22	0.9300
O2—C34	1.4270 (18)	C23—H23	0.9300
O2—C33	1.436 (2)	C24—C25	1.347 (3)
N1—C1	1.389 (2)	C24—C32	1.493 (3)
N1—C8	1.398 (2)	C25—C26	1.424 (3)
N1—C11	1.477 (2)	C25—H25	0.9300
N2—C31	1.394 (2)	C26—C27	1.398 (3)
N2—C24	1.395 (2)	C26—C31	1.407 (2)
N2—C34	1.479 (2)	C27—C28	1.367 (3)
C1—C2	1.352 (3)	C27—H27	0.9300
C1—C9	1.493 (3)	C28—C29	1.390 (3)
C2—C3	1.418 (3)	C28—H28	0.9300
C2—H2	0.9300	C29—C30	1.375 (2)
C3—C4	1.397 (3)	C29—H29	0.9300
C3—C8	1.405 (2)	C30—C31	1.389 (2)
C4—C5	1.363 (3)	C30—H30	0.9300
C4—H4	0.9300	C32—C33	1.498 (3)
C5—C6	1.384 (3)	C32—H32A	0.9700
C5—H5	0.9300	C32—H32B	0.9700
C6—C7	1.379 (2)	C33—H33A	0.9700
C6—H6	0.9300	C33—H33B	0.9700
C7—C8	1.388 (2)	C34—C41	1.525 (2)
C7—H7	0.9300	C34—C35	1.528 (2)
C9—C10	1.500 (3)	C35—C40	1.379 (2)
C9—H9A	0.9700	C35—C36	1.391 (2)
C9—H9B	0.9700	C36—C37	1.378 (2)
C10—H10A	0.9700	C36—H36	0.9300
C10—H10B	0.9700	C37—C38	1.363 (2)
C11—C12	1.531 (2)	C37—H37	0.9300
C11—C18	1.531 (2)	C38—C39	1.355 (2)
C12—C17	1.377 (2)	C39—C40	1.387 (2)
C12—C13	1.388 (2)	C39—H39	0.9300
C13—C14	1.378 (2)	C40—H40	0.9300
C13—H13	0.9300	C41—C46	1.384 (2)
C14—C15	1.363 (3)	C41—C42	1.390 (2)
C14—H14	0.9300	C42—C43	1.382 (2)
C15—C16	1.364 (2)	C42—H42	0.9300
C16—C17	1.391 (2)	C43—C44	1.368 (3)
C16—H16	0.9300	C43—H43	0.9300
C17—H17	0.9300	C44—C45	1.360 (2)
C18—C23	1.383 (2)	C45—C46	1.384 (2)
C18—C19	1.385 (2)	C45—H45	0.9300
C19—C20	1.381 (2)	C46—H46	0.9300
			
C11—O1—C10	113.98 (13)	C23—C22—H22	120.7
C34—O2—C33	114.17 (13)	C18—C23—C22	120.77 (16)
C1—N1—C8	108.31 (14)	C18—C23—H23	119.6
C1—N1—C11	122.61 (14)	C22—C23—H23	119.6
C8—N1—C11	127.83 (13)	C25—C24—N2	109.24 (16)
C31—N2—C24	108.11 (14)	C25—C24—C32	130.84 (18)
C31—N2—C34	128.02 (13)	N2—C24—C32	119.73 (17)
C24—N2—C34	122.53 (14)	C24—C25—C26	108.43 (16)
C2—C1—N1	108.87 (17)	C24—C25—H25	125.8
C2—C1—C9	131.53 (18)	C26—C25—H25	125.8
N1—C1—C9	119.44 (17)	C27—C26—C31	119.42 (17)
C1—C2—C3	108.76 (16)	C27—C26—C25	133.75 (18)
C1—C2—H2	125.6	C31—C26—C25	106.82 (16)
C3—C2—H2	125.6	C28—C27—C26	119.34 (18)
C4—C3—C8	118.98 (18)	C28—C27—H27	120.3
C4—C3—C2	134.24 (18)	C26—C27—H27	120.3
C8—C3—C2	106.78 (16)	C27—C28—C29	120.42 (19)
C5—C4—C3	119.63 (19)	C27—C28—H28	119.8
C5—C4—H4	120.2	C29—C28—H28	119.8
C3—C4—H4	120.2	C30—C29—C28	121.94 (19)
C4—C5—C6	120.60 (19)	C30—C29—H29	119.0
C4—C5—H5	119.7	C28—C29—H29	119.0
C6—C5—H5	119.7	C29—C30—C31	117.86 (17)
C7—C6—C5	121.8 (2)	C29—C30—H30	121.1
C7—C6—H6	119.1	C31—C30—H30	121.1
C5—C6—H6	119.1	C30—C31—N2	131.60 (15)
C6—C7—C8	117.54 (17)	C30—C31—C26	120.99 (16)
C6—C7—H7	121.2	N2—C31—C26	107.39 (14)
C8—C7—H7	121.2	C24—C32—C33	111.25 (16)
C7—C8—N1	131.33 (15)	C24—C32—H32A	109.4
C7—C8—C3	121.39 (16)	C33—C32—H32A	109.4
N1—C8—C3	107.28 (15)	C24—C32—H32B	109.4
C1—C9—C10	111.49 (16)	C33—C32—H32B	109.4
C1—C9—H9A	109.3	H32A—C32—H32B	108.0
C10—C9—H9A	109.3	O2—C33—C32	110.98 (16)
C1—C9—H9B	109.3	O2—C33—H33A	109.4
C10—C9—H9B	109.3	C32—C33—H33A	109.4
H9A—C9—H9B	108.0	O2—C33—H33B	109.4
O1—C10—C9	110.03 (16)	C32—C33—H33B	109.4
O1—C10—H10A	109.7	H33A—C33—H33B	108.0
C9—C10—H10A	109.7	O2—C34—N2	107.28 (13)
O1—C10—H10B	109.7	O2—C34—C41	104.15 (12)
C9—C10—H10B	109.7	N2—C34—C41	110.25 (13)
H10A—C10—H10B	108.2	O2—C34—C35	109.44 (12)
O1—C11—N1	107.82 (12)	N2—C34—C35	111.28 (13)
O1—C11—C12	103.37 (12)	C41—C34—C35	113.99 (13)
N1—C11—C12	110.13 (13)	C40—C35—C36	118.30 (15)
O1—C11—C18	109.93 (13)	C40—C35—C34	123.24 (15)
N1—C11—C18	111.30 (12)	C36—C35—C34	118.45 (14)
C12—C11—C18	113.86 (13)	C37—C36—C35	121.08 (16)
C17—C12—C13	118.51 (15)	C37—C36—H36	119.5
C17—C12—C11	123.30 (15)	C35—C36—H36	119.5
C13—C12—C11	118.12 (14)	C38—C37—C36	118.38 (17)
C14—C13—C12	121.31 (16)	C38—C37—H37	120.8
C14—C13—H13	119.3	C36—C37—H37	120.8
C12—C13—H13	119.3	C39—C38—F3	118.82 (16)
C15—C14—C13	118.20 (17)	C39—C38—C37	122.69 (17)
C15—C14—H14	120.9	F3—C38—C37	118.49 (17)
C13—C14—H14	120.9	C38—C39—C40	118.64 (16)
F1—C15—C14	118.70 (17)	C38—C39—H39	120.7
F1—C15—C16	118.47 (17)	C40—C39—H39	120.7
C14—C15—C16	122.82 (16)	C35—C40—C39	120.88 (16)
C15—C16—C17	118.25 (17)	C35—C40—H40	119.6
C15—C16—H16	120.9	C39—C40—H40	119.6
C17—C16—H16	120.9	C46—C41—C42	118.43 (16)
C12—C17—C16	120.92 (17)	C46—C41—C34	123.08 (15)
C12—C17—H17	119.5	C42—C41—C34	118.47 (15)
C16—C17—H17	119.5	C43—C42—C41	120.93 (17)
C23—C18—C19	118.40 (15)	C43—C42—H42	119.5
C23—C18—C11	122.97 (15)	C41—C42—H42	119.5
C19—C18—C11	118.63 (14)	C44—C43—C42	118.30 (17)
C20—C19—C18	121.29 (16)	C44—C43—H43	120.8
C20—C19—H19	119.4	C42—C43—H43	120.8
C18—C19—H19	119.4	C45—C44—F4	118.61 (17)
C21—C20—C19	118.15 (17)	C45—C44—C43	122.87 (17)
C21—C20—H20	120.9	F4—C44—C43	118.51 (17)
C19—C20—H20	120.9	C44—C45—C46	118.27 (17)
C22—C21—F2	118.94 (17)	C44—C45—H45	120.9
C22—C21—C20	122.65 (17)	C46—C45—H45	120.9
F2—C21—C20	118.41 (18)	C41—C46—C45	121.21 (17)
C21—C22—C23	118.69 (16)	C41—C46—H46	119.4
C21—C22—H22	120.7	C45—C46—H46	119.4
			
C8—N1—C1—C2	−1.1 (2)	C31—N2—C24—C25	−0.7 (2)
C11—N1—C1—C2	−169.22 (15)	C34—N2—C24—C25	−168.44 (15)
C8—N1—C1—C9	−176.91 (16)	C31—N2—C24—C32	−176.31 (16)
C11—N1—C1—C9	15.0 (2)	C34—N2—C24—C32	15.9 (2)
N1—C1—C2—C3	0.7 (2)	N2—C24—C25—C26	0.1 (2)
C9—C1—C2—C3	175.8 (2)	C32—C24—C25—C26	175.05 (19)
C1—C2—C3—C4	179.9 (2)	C24—C25—C26—C27	−178.4 (2)
C1—C2—C3—C8	−0.1 (2)	C24—C25—C26—C31	0.5 (2)
C8—C3—C4—C5	−0.1 (3)	C31—C26—C27—C28	−0.8 (3)
C2—C3—C4—C5	180.0 (2)	C25—C26—C27—C28	178.0 (2)
C3—C4—C5—C6	−0.1 (3)	C26—C27—C28—C29	0.8 (3)
C4—C5—C6—C7	0.7 (3)	C27—C28—C29—C30	0.5 (3)
C5—C6—C7—C8	−1.2 (3)	C28—C29—C30—C31	−1.7 (3)
C6—C7—C8—N1	−178.82 (17)	C29—C30—C31—N2	−176.50 (17)
C6—C7—C8—C3	1.0 (3)	C29—C30—C31—C26	1.7 (3)
C1—N1—C8—C7	−179.14 (18)	C24—N2—C31—C30	179.38 (18)
C11—N1—C8—C7	−11.8 (3)	C34—N2—C31—C30	−13.7 (3)
C1—N1—C8—C3	1.02 (18)	C24—N2—C31—C26	1.02 (18)
C11—N1—C8—C3	168.35 (15)	C34—N2—C31—C26	167.89 (14)
C4—C3—C8—C7	−0.4 (3)	C27—C26—C31—C30	−0.4 (3)
C2—C3—C8—C7	179.56 (16)	C25—C26—C31—C30	−179.52 (16)
C4—C3—C8—N1	179.47 (16)	C27—C26—C31—N2	178.13 (15)
C2—C3—C8—N1	−0.59 (19)	C25—C26—C31—N2	−0.95 (19)
C2—C1—C9—C10	163.8 (2)	C25—C24—C32—C33	164.7 (2)
N1—C1—C9—C10	−21.5 (3)	N2—C24—C32—C33	−20.8 (3)
C11—O1—C10—C9	−66.94 (19)	C34—O2—C33—C32	−66.25 (19)
C1—C9—C10—O1	45.0 (2)	C24—C32—C33—O2	43.6 (2)
C10—O1—C11—N1	56.31 (18)	C33—O2—C34—N2	56.57 (18)
C10—O1—C11—C12	172.92 (14)	C33—O2—C34—C41	173.47 (14)
C10—O1—C11—C18	−65.18 (18)	C33—O2—C34—C35	−64.28 (18)
C1—N1—C11—O1	−30.2 (2)	C31—N2—C34—O2	163.30 (14)
C8—N1—C11—O1	164.11 (14)	C24—N2—C34—O2	−31.53 (19)
C1—N1—C11—C12	−142.33 (15)	C31—N2—C34—C41	50.5 (2)
C8—N1—C11—C12	52.0 (2)	C24—N2—C34—C41	−144.34 (15)
C1—N1—C11—C18	90.43 (17)	C31—N2—C34—C35	−77.02 (19)
C8—N1—C11—C18	−75.26 (19)	C24—N2—C34—C35	88.15 (17)
O1—C11—C12—C17	117.65 (16)	O2—C34—C35—C40	129.85 (16)
N1—C11—C12—C17	−127.39 (16)	N2—C34—C35—C40	11.5 (2)
C18—C11—C12—C17	−1.6 (2)	C41—C34—C35—C40	−114.00 (17)
O1—C11—C12—C13	−59.36 (18)	O2—C34—C35—C36	−49.3 (2)
N1—C11—C12—C13	55.61 (18)	N2—C34—C35—C36	−167.72 (14)
C18—C11—C12—C13	−178.58 (14)	C41—C34—C35—C36	66.83 (19)
C17—C12—C13—C14	−0.3 (2)	C40—C35—C36—C37	0.6 (3)
C11—C12—C13—C14	176.84 (15)	C34—C35—C36—C37	179.83 (16)
C12—C13—C14—C15	0.0 (3)	C35—C36—C37—C38	0.2 (3)
C13—C14—C15—F1	179.12 (16)	C36—C37—C38—C39	−1.0 (3)
C13—C14—C15—C16	0.3 (3)	C36—C37—C38—F3	179.34 (16)
F1—C15—C16—C17	−179.05 (16)	F3—C38—C39—C40	−179.46 (15)
C14—C15—C16—C17	−0.2 (3)	C37—C38—C39—C40	0.9 (3)
C13—C12—C17—C16	0.4 (2)	C36—C35—C40—C39	−0.7 (2)
C11—C12—C17—C16	−176.61 (15)	C34—C35—C40—C39	−179.91 (15)
C15—C16—C17—C12	−0.1 (3)	C38—C39—C40—C35	0.0 (3)
O1—C11—C18—C23	134.65 (16)	O2—C34—C41—C46	122.00 (16)
N1—C11—C18—C23	15.3 (2)	N2—C34—C41—C46	−123.20 (16)
C12—C11—C18—C23	−109.93 (17)	C35—C34—C41—C46	2.8 (2)
O1—C11—C18—C19	−45.0 (2)	O2—C34—C41—C42	−56.34 (18)
N1—C11—C18—C19	−164.36 (15)	N2—C34—C41—C42	58.46 (18)
C12—C11—C18—C19	70.44 (19)	C35—C34—C41—C42	−175.54 (14)
C23—C18—C19—C20	1.2 (3)	C46—C41—C42—C43	−0.5 (2)
C11—C18—C19—C20	−179.11 (16)	C34—C41—C42—C43	177.87 (15)
C18—C19—C20—C21	0.2 (3)	C41—C42—C43—C44	0.1 (3)
C19—C20—C21—C22	−1.5 (3)	C42—C43—C44—C45	0.3 (3)
C19—C20—C21—F2	178.64 (16)	C42—C43—C44—F4	179.35 (16)
F2—C21—C22—C23	−178.82 (16)	F4—C44—C45—C46	−179.18 (15)
C20—C21—C22—C23	1.3 (3)	C43—C44—C45—C46	−0.1 (3)
C19—C18—C23—C22	−1.4 (3)	C42—C41—C46—C45	0.7 (2)
C11—C18—C23—C22	178.95 (15)	C34—C41—C46—C45	−177.62 (15)
C21—C22—C23—C18	0.2 (3)	C44—C45—C46—C41	−0.4 (3)

### Hydrogen-bond geometry (Å, °)

**Table:**

*D*—H···*A*	*D*—H	H···*A*	*D*···*A*	*D*—H···*A*
C14—H14···Cg3^i^	0.93	2.88	3.769 (2)	159
C29—H29···Cg5^ii^	0.93	2.93	3.747 (2)	148
C43—H43···Cg11^iii^	0.93	2.79	3.695 (2)	165
C40—H40···Cg9	0.93	2.74	3.3790 (7)	127

Symmetry codes: (i) −*x*+1, −*y*, −*z*+1; (ii) *x*−1, *y*, *z*; (iii) −*x*, −*y*+1, −*z*.
